# An immune-related nomogram model that predicts the overall survival of patients with lung adenocarcinoma

**DOI:** 10.1186/s12890-022-01902-6

**Published:** 2022-03-30

**Authors:** Jing Sun, Yan Yan, Yiming Meng, Yushu Ma, Tianzhao Du, Tao Yu, Haozhe Piao

**Affiliations:** 1 Central Laboratory, Cancer Hospital of China Medical University, Liaoning Cancer Hospital and Institute, Shenyang, 110042 Liaoning Province People’s Republic of China; 2 Department of Medical Imaging, Cancer Hospital of China Medical University, Liaoning Cancer Hospital and Institute, Shenyang, 110042 Liaoning Province People’s Republic of China; 3 Department of Neurosurgery, Cancer Hospital of China Medical University, Liaoning Cancer Hospital and Institute, Shenyang, 110042 Liaoning Province People’s Republic of China

**Keywords:** Lung adenocarcinoma, Immunophenotype, Survival prediction, Nomogram model, TCGA database

## Abstract

**Background:**

Lung adenocarcinoma accounts for approximately 40% of all primary lung cancers; however, the mortality rates remain high. Successfully predicting progression and overall (OS) time will provide clinicians with more options to manage this disease.

**Methods:**

We analyzed RNA sequencing data from 510 cases of lung adenocarcinoma from The Cancer Genome Atlas database using CIBERSORT, ImmuCellAI, and ESTIMATE algorithms. Through these data we constructed 6 immune subtypes and then compared the difference of OS, immune infiltration level and gene expression between these immune subtypes. Also, all the subtypes and immune cells infiltration level were used to evaluate the relationship with prognosis and we introduced lasso-cox method to constructe an immune-related prognosis model. Finally we validated this model in another independent cohort.

**Results:**

The C3 immune subtype of lung adenocarcinoma exhibited longer survival, whereas the C1 subtype was associated with a higher mutation rate of *MUC17* and *FLG* genes compared with other subtypes. A multifactorial correlation analysis revealed that immune cell infiltration was closely associated with overall survival. Using data from 510 cases, we constructed a nomogram prediction model composed of clinicopathologic factors and immune signatures. This model produced a C-index of 0.73 and achieved a C-index of 0.844 using a validation set.

**Conclusions:**

Through this study we constructed an immune related prognosis model to instruct lung adenocarcinoma’s OS and validated its value in another independent cohost. These results will be useful in guiding treatment for lung adenocarcinoma based on tumor immune profiles.

**Supplementary Information:**

The online version contains supplementary material available at 10.1186/s12890-022-01902-6.

## Introduction

Lung cancer is the most common malignant tumor, the leading cause of cancer-related deaths in men, and the second leading cause in women. Lung adenocarcinoma (LUAD) is a subtype of non-small cell lung cancer which accounts for 30–40% of all lung cancers [1, 2]. The underlying molecular mechanisms of lung carcinogenesis have been investigated by studying mutations, chromosomal dislocation and loss, epigenetic modifications, signaling pathways, and therapeutic interventions [3–7]. However, it is difficult to determine which factors promote the onset and progression of the disease and which are downstream secondary events caused by the tumor.

In recent decades, various metadata, including transcriptome sequencing, whole exome sequencing, epigenetic modification, pathology, and imaging data from patients of different races, geographic regions, and tumor types, have become available in public databases. These data provide an opportunity for exploration of the molecular mechanisms of tumorigenesis and development. Another important medical advancement has been the discovery of immune surveillance sites. Surveillance site-related proteins such as programmed cell death protein 1 (PD-1), programmed death-ligand 1 (PD-L1), and cytotoxic T-lymphocyte-associated protein 4 are described as modulators of immune function as they can transform the host immune response from immunosuppressive to an activated state by immunotherapy [8–10]. In 2019, Wu et al. reported that anti-PD-1 and anti-PD-L1 antibodies could benefit patients with non-small cell lung cancer, and that an anti-PD-1 monoclonal antibody combined with chemotherapy effectively prolonged disease-free survival (DFS) and overall survival (OS) provided that the PD-1/PD-L1 antibody positivity rate was > 1% [11–13]. As a result, these antibodies have been recommended as a first-line treatment for non-small cell lung cancer. Additionally, they were shown to provide a survival benefit for patients with melanoma, renal cell carcinoma, and colorectal cancer [14–16].

The response to immunotherapy primarily depends on immunomodulatory factors such as immune cell infiltration, mutation load, the tumor:mesenchyme ratio, tumor elasticity, and stiffness within the tumor microenvironment. Therefore, neither the infiltration of a certain immune cell subset, nor the activation or inhibition of a particular signaling pathway alone is sufficient to evaluate the immune status or predict disease progression. In 2018, Chandra et al. incorporated transcriptome sequencing results from more than 11,000 cases representing 33 solid tumors, except for hematologic tumors, and proposed an immunological classification for different tumor types. This new method did not take into account the tissue origin or anatomical site of the tumor, but instead classified tumors into six immune subtypes based on their immunological characteristics: C1 (wound-healing type), C2 (interferon [IFN]-γ type), C3 (infected type), C4 (immuno-deleted type), C5 (immuno-resting type), and C6 (transforming growth factor [TGFβ] type) [17]. The results indicated that the immunophenotype could predict the risk of tumor recurrence and metastasis better than the traditional method of tumor–node–metastasis (TNM) staging [18–20].

In the present study, immunotyping was performed for 510 cases of LUAD together with transcriptome sequencing data from The Cancer Genome Atlas (TCGA) library. The data were analyzed with respect to differences in immune cell infiltration, mutation load, and the tumor:mesenchyme ratio of the different subtypes. A nomogram prediction model for OS was established with a C-index of 0.73 in the training group and a C-index of 0.84 in the validation group. This model may enable physicians to predict the clinical outcome of patients with LUAD and to guide disease management.

## Materials and methods

### Immunophenotyping of LUAD cases using the CIBERSORT algorithm

Data for 510 LUAD patients together with RNA sequencing data and corresponding clinical information were obtained from the TCGA database (https://www.cbioportal.org/). Then raw expression matrix data downloaded from cBioportal was uploaded to CRIiAtlas website for immune subtype analysis (https://isb-cgc.shinyapps.io/shiny-iatlas/) [21], the online tool can identify 6 immune subtypes across pan-cancer. Then, the log-rank test was performed to evaluate differences in DFS and OS between different subtypes of LUAD. Finally, differences in T, N, and M stages among diverse immune subtypes were analyzed by the Chi-square test in R [22].

### Enrichment analysis of differentially expressed genes for different subtypes of LUAD

The DESeq2 algorithm (R package version 1.28.1) was used to analyze DEGs for different subtypes of LUAD using default parameters. Those with a false discovery rate ≤ 0.05 and log2FC > 1.5 were grouped as DEGs [23]. To investigate biological functions, the clusterProfiler module of the R package was used to perform gene ontology enrichment analysis of the top 200 DEGs.

### Analyzing differences in the mutation load of different immune subtypes of lung adenocarcinoma

Of the 510 available lung adenocarcinoma samples we can downloaded their transcriptome sequencing data, copy number aberation data, and clinical data, but there are only 227 samples had total exome sequencing data. Therefore, we analyzed the mutation load of these 227 cases. We selected eight core genes (EGFR, ALK, KRAS, BRAF, MET, ERBB2, RET, and ROS1) associated with non-small cell lung cancer, as recommended by National Comprehensive Cancer Network guidelines [24], and 14 genes exhibiting the highest mutation frequency in the 227 LUAD cases for subsequent analysis. The types of mutations analyzed included point mutations (missense mutations), insertions or deletions (indels), early terminations (stop-gain mutations), frameshift mutations (frameshifts), and alternative splicing mutations (splice site mutations). The Chi-square test was used to analyze differences in mutation detection rates among the different immune subtypes.

### Analyzing the correlation between 24 immune cell infiltrations with OS and DFS using the ImmuneCellAI algorithm

ImmuCellAI (website tool http://bioinfo.life.hust.edu.cn/ImmuCellAI#!/), a deconvolution algorithm was used to estimate the percentage of different immune cell subsets in each sample [25]. This deconvolution algorithm was used to simultaneously determine the composition of 24 cell subsets in each sample using RNAseq data [25]. The cells included 17 T cell subsets: CD4^+^ T cells, CD8^+^ T cells, naïve CD4^+^ T cells, naïve CD8^+^ T cells, central memory T cells, effector memory T cells, regulatory T cells (induced regulatory T cells and natural regulatory T cells), mucosal-associated invariant T cells (which function in both the inflammatory response and in maintaining homeostasis), T regulatory type 1 cells (Tr1 cells, which down-modulate immune responses through production of the immunosuppressive cytokines interleukin-10 and TGF-β), T helper (Th)1 cells, Th2 cells, Th17 cells, follicular auxiliary T cells (which play a key role in T cell-dependent B cell responses), cytotoxic T cells, exhausted T cells, γδT cells, and natural killer T cells. There were also six important effector B cells: macrophages, monocytes, neutrophils, dendritic cells, and natural killer (NK) cells.

We classified the samples into high- and low-level expressing groups according to their median score. Samples with values above the median were considered high expressors, and those with values below were identified as low expressors. We analyzed the correlation between the immune cell infiltrate and the DFS and OS with the log-rank test, with *p* < 0.05 considered statistically significant. The Wilcoxon test was used to analyze the characteristics of immune cell distribution among different immune cell subtypes. Cox multifactorial regression analysis was used to evaluate the correlation between infiltrating immune cells and OS in patients with LUAD.

### Analyzing differences in the tumor: mesenchyme ratio of different subtypes of LUAD using the ESTIMATE algorithm

According to the ESTIMATE algorithm, they defined a signature containing 141 genes which represented mesenchyme cell characteristics and using single sample gene enrichment analysis (ssGSEA) to determine the enrichment of mesenchyme cells in tumor samples [26]. The expression profiles of these genes were analyzed in its R package (version 0.30.0) and we got two score of mesenchyme content and tumor purity of each sample. Larger scores representing a higher mesenchyme content and lower tumor purity. Finally, the log-rank test was used to analyze the correlation between tumor:mesenchyme ratio scoring and DFS and OS.

### Establishing and verifying the OS prediction model for LUAD

Initially, LUAD cases in the TCGA database were regarded as the training group. Step-wise regression was introduced for feature selection, then we constructed a prognosis model using the coxph function in the R survival package (version 3.2.7). Next, we established the nomogram OS evaluation model, which required a C-index of 0.7 or higher. The model was calibrated using the dataset of studies published on top of the cell as a validation set (GSE140343) [27]. The standard curves for 1-, 3-, and 5-year survival were plotted separately and analyzed to determine whether the nomogram assessment model was consistent with the actual survival time. Subsequently, we divided the training and validation data into low- (score < 0) and high-risk (score > 0) groups based on the nomogram model. Finally, the log-rank test was used to determine whether there was a significant difference in OS between the two groups.

## Results

### Immunotyping of LUAD using the CIBERSORT algorithm

**Fig. 1:**
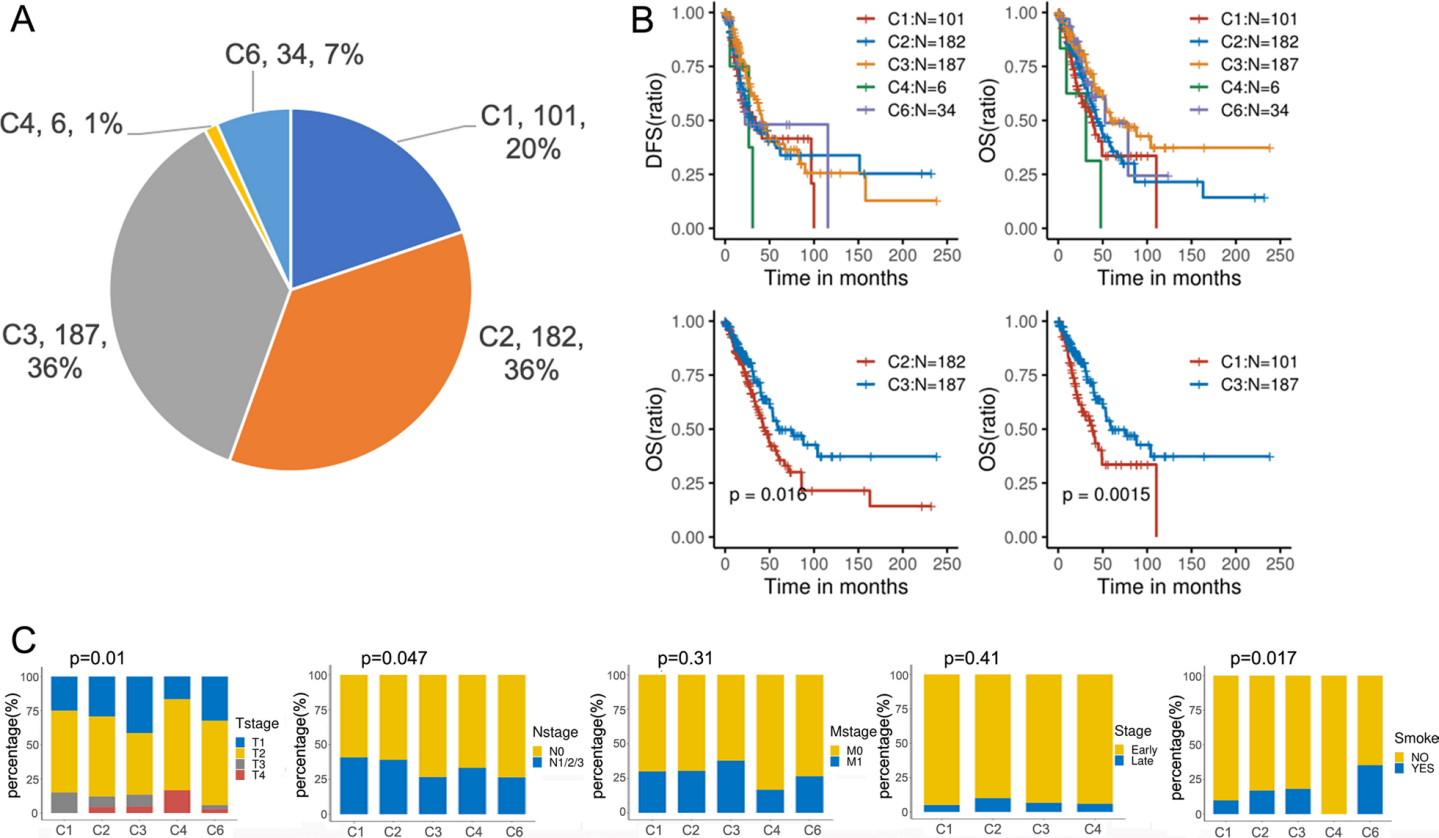
Immunoclassification of patients with lung adenocarcinoma (LUAD) in TCGA. **A** Based on RNAseq data, the immune subtypes of 510 patients with LUAD data were analyzed using the CIBERSORT algorithm in TCGA. The percentage of different immune subtypes is shown in the pie chart. **B** Kaplan–Meier analysis of overall survival in LUAD patients with different immune subtypes. **C** Analysis of the differences between TNM stage and immune subtypes by Fisher’s test

The clinicopathological characteristics of the 510 LUAD cases from the TCGA database are provided in Table 1. The CIBERSORT algorithm was used to immunophenotype the transcriptome sequencing data of these cases. As shown in Fig. 1A, the C1 subtype accounted for 20% (trauma repair type, 101/510), the C2 subtype for 35.7% (IFNγ type, 182/510), the C3 subtype for 36.6% (infectious type, 187/510), the C4 subtype for 1.17% (lymph node deletion type, 6/510), and the C5 subtype for 5.6% (TGFβ-dominant type). From this analysis, the proportions of C2 and C3 subtypes were highest, with both exceeding 35% of the total, whereas the C4 subtype exhibited only a small fraction and the C5 subtype was not represented. Log-rank test analysis of the differences in DFS and OS among the different subtypes indicated that the C3 subtype was associated with a significantly higher OS than other subtypes, whereas there was no significant difference in DFS between subtypes (Fig. 1B). As the C1, C2, and C3 subtypes accounted for 92.3% of the total, we next focused on the differences in mutation load, immune cell infiltration, and the tumor:mesenchyme ratio among these subtypes.

**Table 1:** Clinicopathologic features of patients with LUAD in the TCGA

Characteristic	Varl	Freq
Gender	Female	**274 (53.73)**
	Male	**236 (46.27)**
Stage	Early	**465 (91.18)**
	Late	**37 (7.25)**
	NA	**8 (1.57)**
Smoke	NO	**423 (82.94)**
	YES	**87 (17.06)**
Tstage	T1	**167 (32.75)**
	T2	**275 (53.92)**
	T3	**46 (9.02)**
	T4	**19 (3.73)**
	NA	**3 (0.59)**
Nstage	N0	**1 (0.2)**
	N1	**328 (64.31)**
	N2	**96 (18.82)**
	N3	**73 (14.31)**
	NX	**2 (0.39)**
Mstage	M0	361 (70.78)
	M1	**10 (1.96)**
	MX	**5 (0.98)**
	NA	**140 (27.45)**

Early means stage I/II and Late means stage III /IV, NA means no information available, NX and MX not evaluated N and M

### Analyzing clinicopathological factors and gene enrichment differences in different subtypes of LUAD

The correlation between clinicopathological factors and immune subtypes was analyzed by Fisher’s test. As shown in Fig. 1C, the proportion of T1 and N0 stages was significantly higher in the C3 subtype compared with C1 and C2 subtypes. We also observed a significantly greater distribution of T1 and N0 stages in the C2 subtype compared with the C1 subtype, whereas smoking was significantly correlated with occurrence of the C6 subtype.

**Fig. 2:**
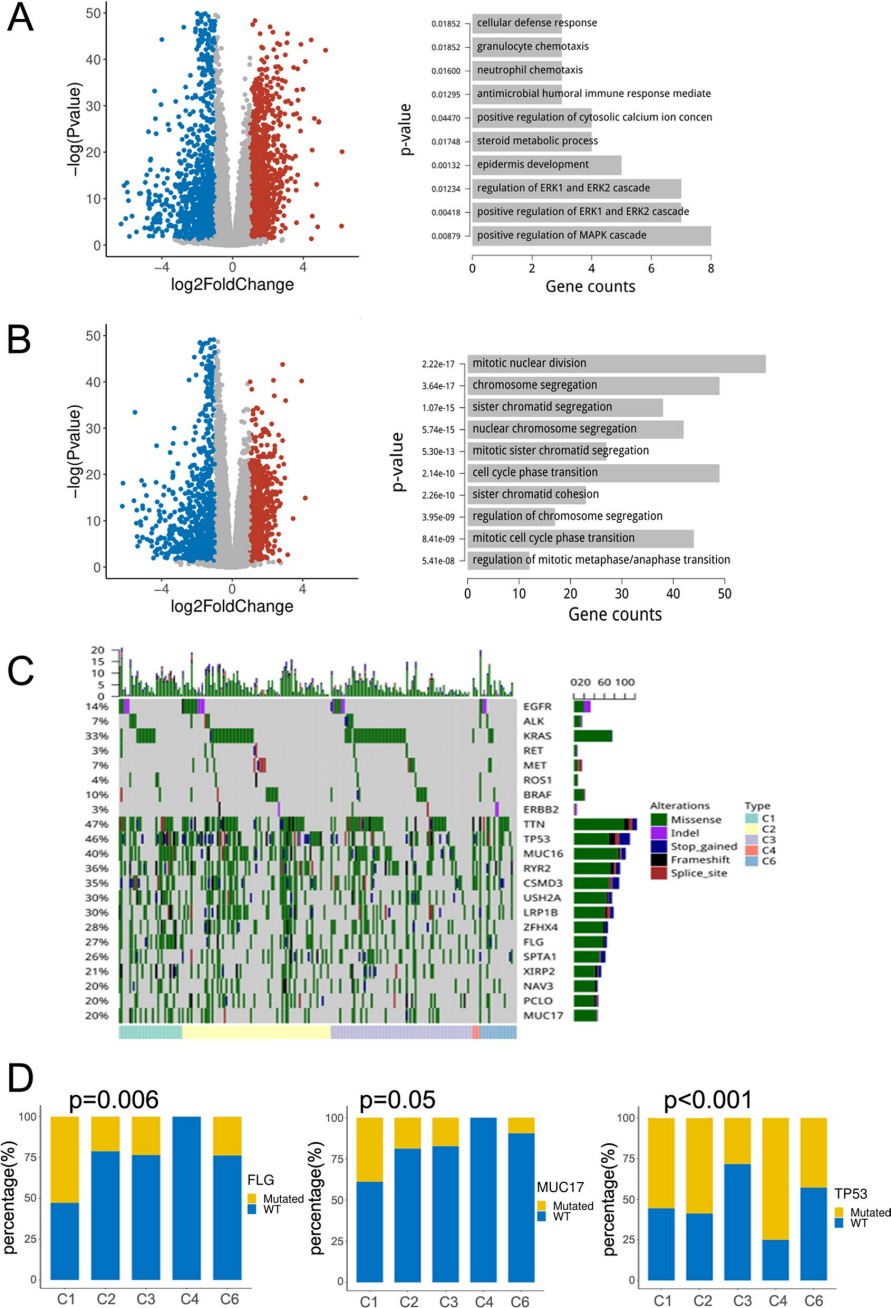
Differentially expressed genes (DEGs) identified in C1 and C3, or C2 and C3 subtypes of LUAD. **A** Volcano map of DEGs for C1 and C3 subtypes. DEGs were obtained by the DESeq2 algorithm (false discovery rate ≤ 0.05 and log2 fc > 1.5). Red dots indicate upregulated genes and blue dots indicate downregulated genes (left). Functional and pathway analyses were performed using the R-package clusterProfiler module (right). **B** Enrichment analysis of DEGs in C2 and C3 subtypes. **C** The mutation load among different immune subtypes in LUAD as analyzed by the Chi-square test. Rows represent genes, and columns represent samples. The intersection represents the sample mutation, the color represents the mutation type, and the color at the bottom represents immune typing. **D** Analysis of differences in *FLG*, *MUC17*, and *p53* mutations among different subtypes of LUAD

Differential gene expression between C1/C3 subtypes and C2/C3 subtypes was analyzed by the DESeq2 algorithm, with a false discovery rate ≤ 0.05 and log2FC > 1.5 as screening criteria. More than 1000 DEGs were identified, of which 500 were upregulated and downregulated simultaneously. Enrichment analysis of the 200 genes exhibiting the most significant differences in expression indicated that genes differentially expressed between C1 and C3 subtypes were mainly associated with extracellular signal‑regulated protein kinase (ERK)1, ERK2, and mitogen-activated protein kinase (MAPK) signaling pathways, epidermal genesis, and steroid metabolism (Fig. 2A). Genes differentially expressed between C2 and C3 subtypes were primarily associated with chromatin segregation and cell cycle transition (Fig. 2B).

### Analyzing differences in mutation load of the different subtypes of LUAD

We next analyzed mutations in eight non-small cell lung cancer core genes recommended by National Comprehensive Cancer Network guidelines and 14 genes with the highest mutation frequency in 510 LUAD cases for which exome sequencing, RNA sequencing, and copy number data were available. The top five most frequently mutated genes were *TTN* (47.1%), *TP53* (46.7%), *MUC16* (40.0%), *RYR2* (36.1%), and *CSMD3* (35.7%; Fig. 2C). Additionally, we found that the mutation detection rate of *FLG* and *MUC17* genes in the C1 subtype was significantly higher than in other subtypes (Fig. 2D). Therefore, the higher mutation load and the activation of ERK1, ERK2, and MAPK signaling pathways were considered potential contributors to the poor prognosis associated with the C1 subtype. The lowest mutation rate was observed for *TP53* in the C3 subtype (Fig. 2D), which is consistent with its better prognosis.

### Analyzing the relationship between immune cell infiltration and OS and DFS of LUAD

**Fig. 3:**
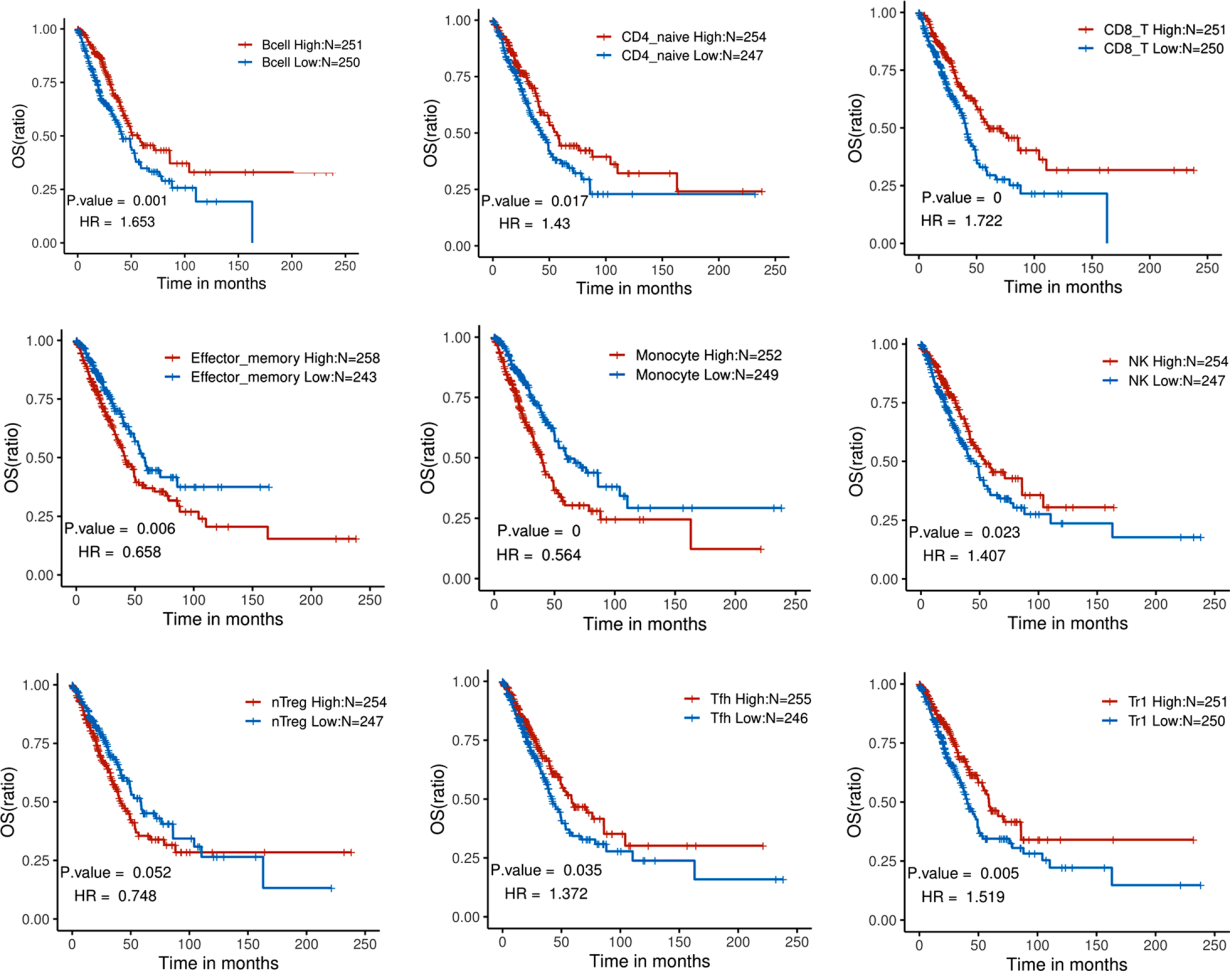
Analysis of the correlation between immune cell infiltration and OS in patients with LUAD. LUAD cases were divided into high and low expression groups based on the median value of immune cell infiltration. The log-rank test assessed the correlation between immune cell infiltration and OS

**Fig. 4:**
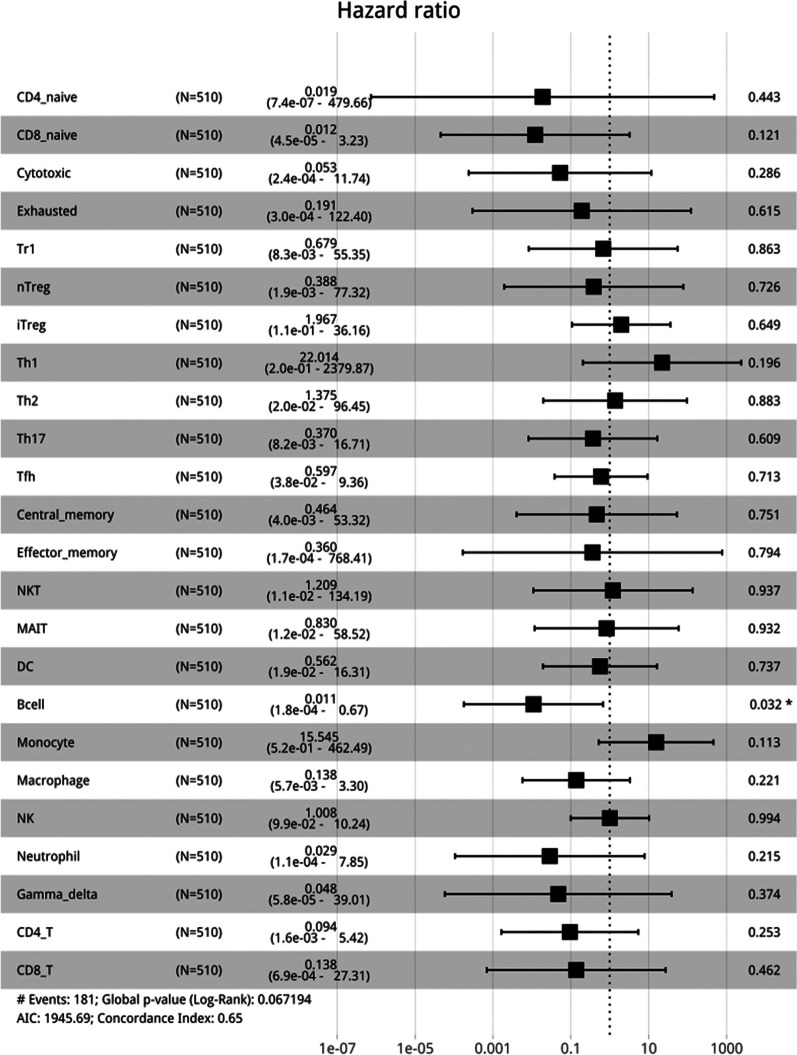
Multifactorial regression analysis evaluating the correlation between immune cell infiltration and OS in patients with LUAD

Using the log-rank test, we found that the infiltration of immune cell subsets was significantly positively correlated with OS (*p* < 0.05), whereas the infiltration of monocytes was significantly negatively correlated (*p* < 0.0001; Fig. 3). Additionally, monocyte infiltration was significantly associated with DFS (*p* = 0.0024) (Attached Fig. 1). Cox multifactorial regression analysis revealed that naïve CD4^+^ T cells, naïve CD8^+^ T cells, B cells, neutrophils, and γδ T cell subsets were significantly correlated with OS (*p* = 0.019, 0.012, 0.011, 0.029, and 0.048, respectively; Fig. 4).

**Fig. 5:**
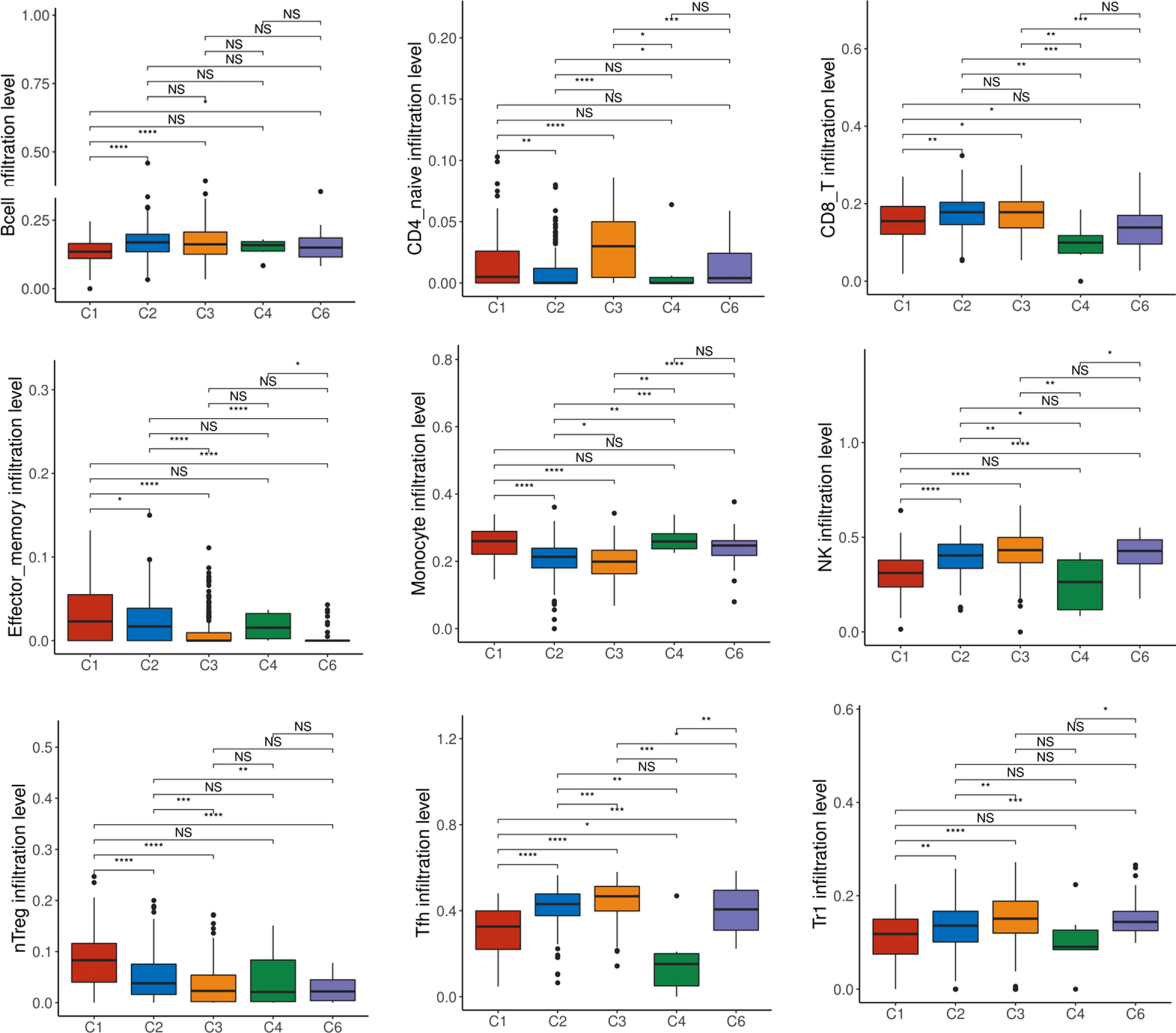
Analysis of the differences in immune cell infiltration in different immune subtypes of LUAD by ImmuCellAI. The Wilcoxon test analyzed the differences between immune cell infiltration and immune subtypes, *means *p* < 0.05, **means *p* < 0.01, ****means *p* < 0.0001

Next, we determined whether the distribution of immune cell subsets varied among the different subtypes of LUAD. As shown in Fig. 5 and Additional file 1: Fig. 3, the infiltration of naïve CD4^+^ T cells, CD8^+^ T cells, CD4^+^ T cells, NK cells, follicular auxiliary T cells, Tr1 cells, and mucosal-associated invariant T cells in the C3 subtype was significantly higher compared with the other subtypes, while the infiltration of natural regulatory T cells and monocytes was significantly lower compared with that in other subtypes. In summary, the distribution characteristics of the immune cells of subtype C3 are empirically consistent with its survival advantage.

### Analyzing differences in the tumor: mesenchyme ratio between different immune subtypes of tumor cells

**Fig. 6:**
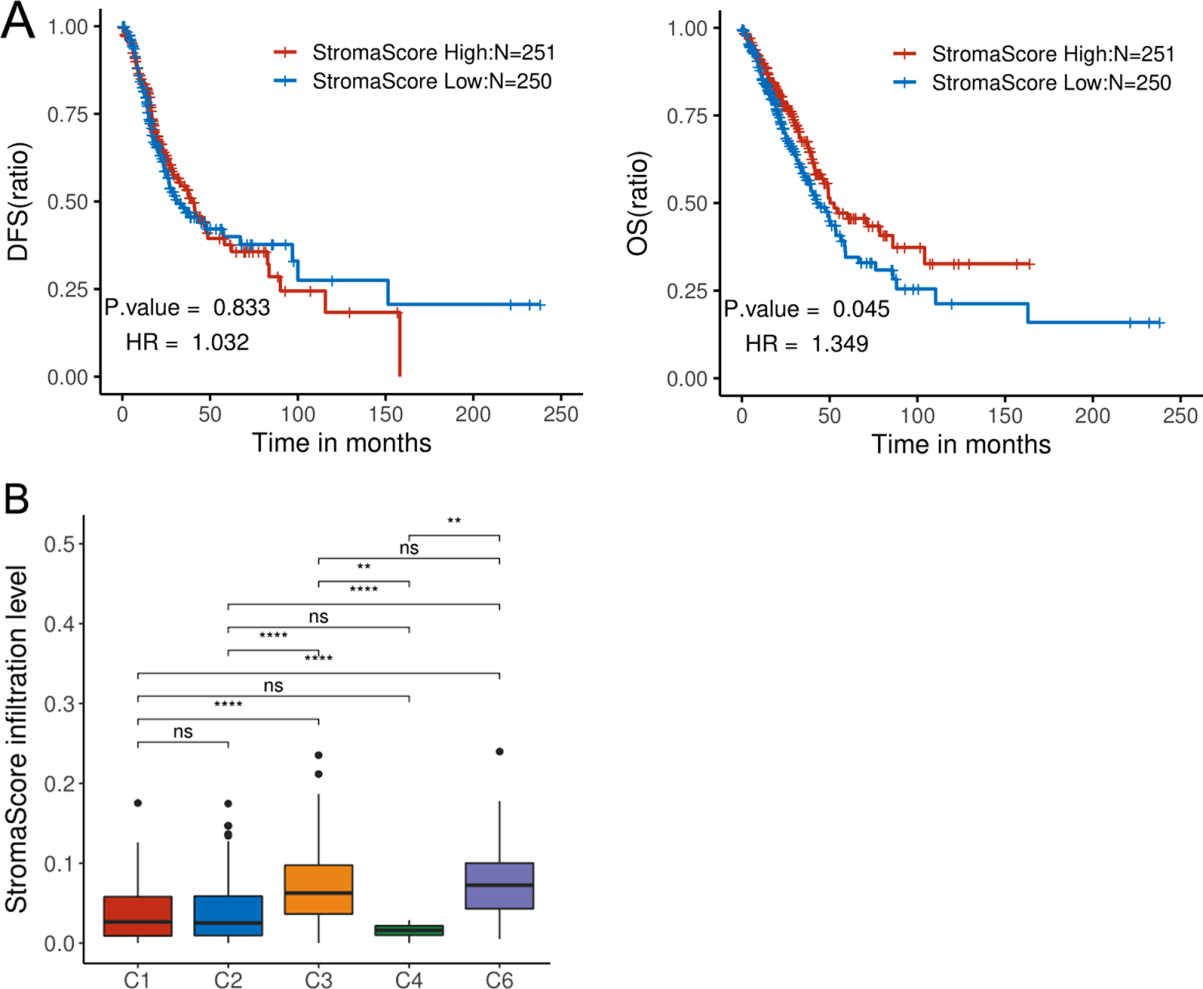
Analysis of the differences in interstitial scores of LUAD using the ESTIMATE algorithm. **A** LUAD cases were divided into high and low stromal groups according to the median value of interstitial scores. The log-rank test assessed the correlation between stromal scores, DFS, and OS. **B** The Wilcoxon test analyzed the differences between interstitial scores in different LUAD immune subtypes

The ESTIMATE algorithm was used to evaluate the tumor:mesenchyme ratio in different subtypes of LUAD, with scoring ranging from 0.000 to 0.2397. The log-rank test revealed that the OS of the highest scoring group was significantly longer than that of the lowest scoring group (*p* = 0.045), and that there was a significant difference between the tumor:mesenchyme ratio and DFS (Fig. 6A). The Wilcoxon test confirmed that the C4 subtype with poor prognosis had the lowest tumor:mesenchyme ratio and the C6 subtype the highest, and that the C3 subtype had a significantly higher tumor:mesenchyme ratio than C2 and C4 subtypes (Fig. 6B). Correlation analysis of the association between the tumor:mesenchyme ratio and immune cell infiltrate found no significant correlation (correlation coefficient < 0.5; Additional file 1: Fig. 2). In summary, significant correlation was detected between the tumor:mesenchyme ratio and OS of LUAD.

### Establishing a nomogram model to predict OS for LUAD

**Fig. 7:**
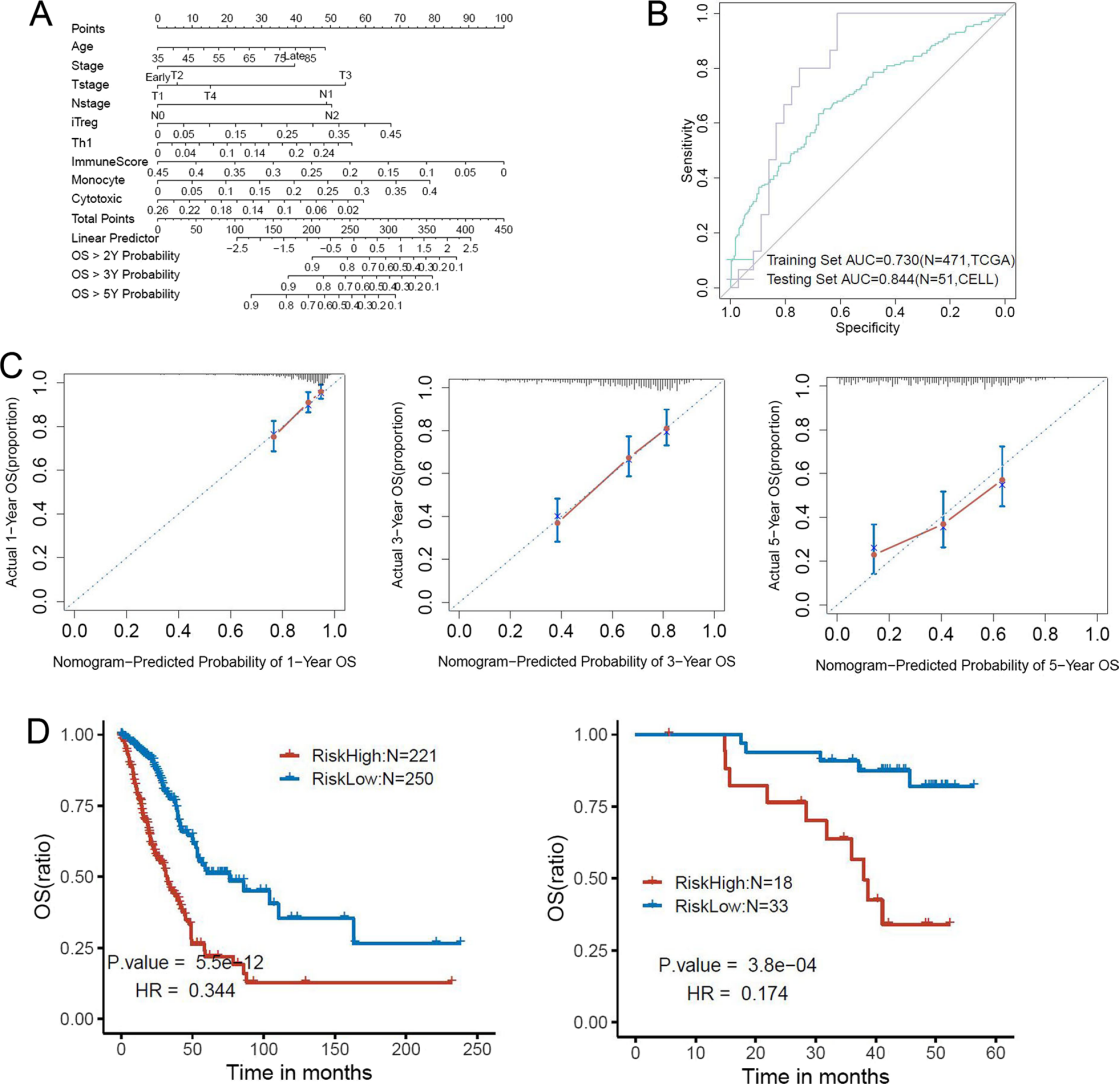
Construction and evaluation of immune-related nomogram model to predict overall survival of patients with LUAD. **A** Using stepwise regression, nine prognostic factors associated with clinical factors, immune subtypes, and immune cell infiltration were included in the nomogram model. **B** The C-index of training set was 0.73 and the C-index of testing group was 0.84. **C** The calibration curve of the nomogram in the training and testing cohort. The estimated by the nomogram is shown on the x-axis, whereas actual survival is shown on the y-axis. The one-year OS (left), three-year OS (middle), five-year OS (right) are shown. **D** The training and testing cohorts are presented as nomogram score. Kaplan–Meier curves were generated to analyze the difference in OS between high- and low-risk groups (with zero as the cut-off point)

The nomogram model was established to analyze the OS of LUAD. The covariates of this model consisted of age, immunological scoring, T/N staging, and immune cells such as Tr1 cells, Th1 cells, and induced regulatory T cells (Fig. 7A). The C-index of the training group, which was composed of TCGA data, reached 0.730. Next, we validated the nomogram model using RNAseq data from 51 lung adenocarcinoma cases described in a recent publication whose clinicopathological characteristics are shown in Table 2 [27]. The C-index of the validation group reached 0.844 (Fig. 7B) and the survival curves at 1, 3, and 5 years after treatment confirmed that the OS values measured by the nomogram model were consistent with the actual OS (Fig. 7C). Finally, we scored the nomogram for the training and validation sets, which was used as a basis to divide the cases into low-risk (score < 0) and high-risk (score > 0) groups. Median OS values were 32.1 months versus 76.2 months for the training group and 38 months versus “not reached” for the validation group. The difference in OS between low- and high-risk groups was significant (*p* < 0.0001 and *p* = 0.00038; Fig. 7D).

**Table 2:** Clinicopathologic features of the LUAD validation group

Characteristic	Varl	Freq
Age	Range	26–76
	Median	60
Gender	Male	17 (33.33)
	Female	34 (66.67)
Smoke	Yes	11 (21.56)
	No	40 (78.43)
Tstage	T1	10 (19.60)
	T2	28 (54.90)
	T3	9 (17.64)
	T4	4 (7.84)
Nstage	N0	29 (56.86)
	N1	6 (11.76)
	N2	16 (31.37)
ImmuneSubtype	C1	0 (0)
	C2	34 (66.67)
	C3	13 (25.49)
	C4	3 (5.88)
	C6	1 (1.96)

## Discussion

The tumor microenvironment acts to nurture and promote tumorigenesis. Factors such as the immune infiltrate, surveillance-related proteins, the tumor:mesenchyme ratio, and trophoblastic vessels determine whether this microenvironment inhibits or promotes tumor growth. In recent years, the application of multidisciplinary techniques to medical research has yielded profound results. In the present study, LUAD cases were classified according to their immunological signatures and analyzed with respect to clinicopathological characteristics for the different subtypes. We found that the C3 subtype was associated with an earlier T and N stage, whereas a positive smoking status was more likely to be seen in patients with the C6 subtype. Gene enrichment analysis revealed a significant association with ERK and MAPK signaling pathways for the C1 subtype and dysregulation of gene expression associated with chromatin segregation and cell cycle transition for the C2 subtype. These results provide novel insights into LUAD that may be applicable for new treatment strategies. These may include targeting ERK or MAPK signaling pathways to treat C1 subtype LUAD, or using inhibitors of cell proliferation for the C2 subtype. Therefore, the results obtained from our analyses of metadata are valuable for future in vitro and in vivo studies.

Previous research has largely focused on the influence of chemical factors on tumor growth, such as the activation of signaling pathways and the expression of chemokines. However, the effect of physical factors on tumor progression should not be overlooked. For example, fibroblasts in the tumor mesenchyme can produce large quantities of elastic fibers and fibrinogen. The resulting sticky and dense mesenchyme may block immune cells from congregating around the tumor and inhibit the migration of immune cells to the tumor bed, despite the persistence of chemotactic signals [28–30]. Histopathological analyses have indicated that a tumor:mesenchyme ratio approximating 1:1 is associated with an improved prognosis compared with other ratios [31, 32]. In this study, we found that the C6 subtype exhibited the highest tumor:mesenchyme ratio, whereas the lowest ratio was seen in the C4 subtype, which had the worst prognosis. However, all tumor:mesenchyme ratios were low in this study, which could be explained by the early entry criteria set by the TCGA, which requires more than 70–80% of tumor tissue to be confirmed by pathology. Hence, our observed correlation between the immune cell infiltrate and tumor:mesenchyme ratio may be biased compared with the actual disease profile.

We used ImmuCellAI bioinformatics analysis to show that the infiltration of naïve CD4^+^ T cells, Tr1 cells, natural regulatory T cells, follicular auxiliary T cells, effector memory T cells, NK cells, CD8^+^ T cells, and B cells was significantly correlated with OS. In the C3 subtype exhibiting the best prognosis, cell subsets contributing positively to an immune response included CD8^+^, CD4^+^, and naïve CD4^+^ T cells, as well as B cells, which were detected at significantly higher levels compared with other subsets. Because there have been some inconsistencies regarding the correlation of NK cells with OS in LUAD and whether it has a positive impact, additional histological experiments will be needed to resolve this issue. Recently, Helmink et al. confirmed from single-cell sequencing data that the proportion of B cells infiltrating tertiary lymphoid tissues in melanoma and renal cell carcinoma was significantly associated with the efficacy of immunotherapy [33]. This is consistent with the positive correlation that we observed between B cell infiltration and OS in LUAD.

When establishing the nomogram model, we initially included many factors such as mutation and tumor:mesenchyme ratio to obtain a good C-index, but the area under the curve values for the validation group were not satisfactory. Therefore, we included immunological factors as the main covariates and subsequently obtained an improved nomogram model which resulted in a satisfactory validation outcome for a small number (n = 53) of cases. The predicted 1- and 3-year survival times for LUAD were also consistent with actual survival times, and the difference in OS between low- and high-risk groups was significant (*p* < 0.0001). Therefore, these results suggest the possibility of predicting OS for patients diagnosed with early-stage LUAD based on their clinicopathological and immunological profiles. Our study could provide clinicians with a new method for predicting OS in LUAD and help guide treatment decisions for patients based on tumor immune profiles.

## Supplementary Information

**Additional file 1.** Supplementary material.

## Data Availability

The data used to support the findings of this study have been downloaded from cBioportal and GSE140343.
