# Supplementary material for: An immune-related nomogram model that predicts the overall survival of patients with lung adenocarcinoma
Source: BMC Pulm Med. 2022 Mar 30;22:114. doi: 10.1186/s12890-022-01902-6 (PMC8969384; doi:10.1186/s12890-022-01902-6)

**Supplementary Materials**

Fig.1. Analyze the correlation of immune cell infiltration and DFS.

All LUAD cases were divided into high and low immune cell infiltration groups by the median value. The log-rank test was used to analyze the correlation between immune cell infiltration and DFS in patients with LUAD.


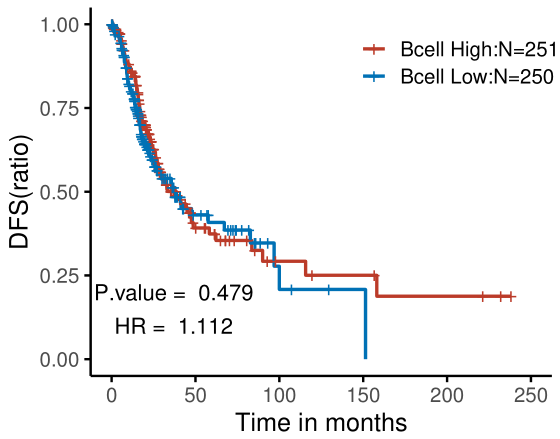

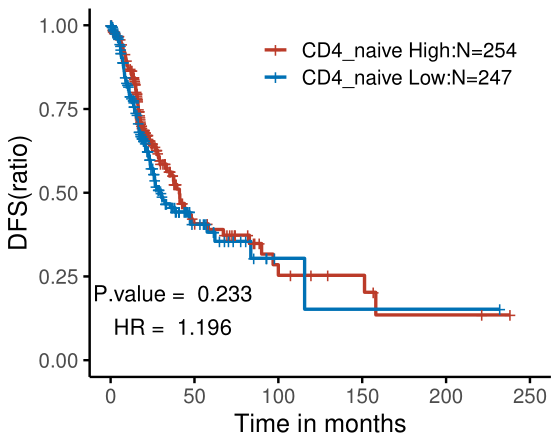


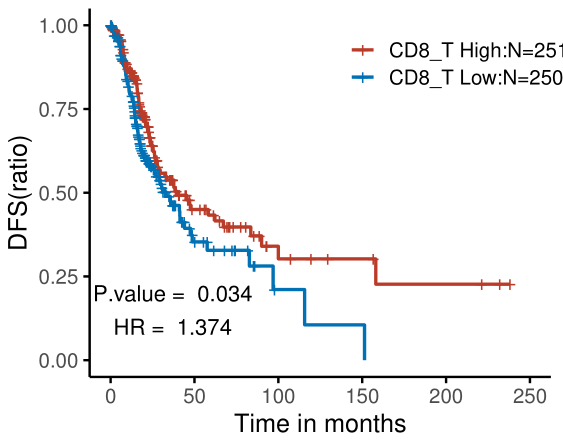

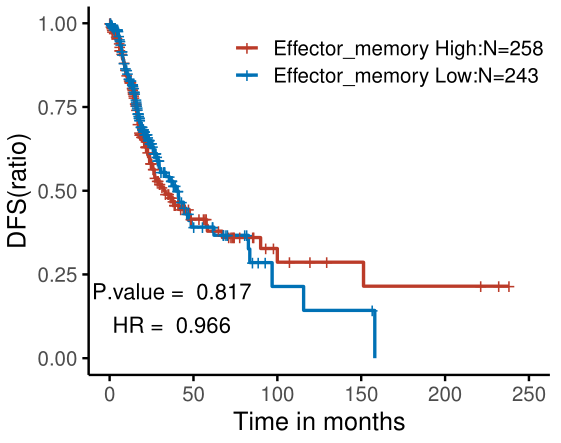


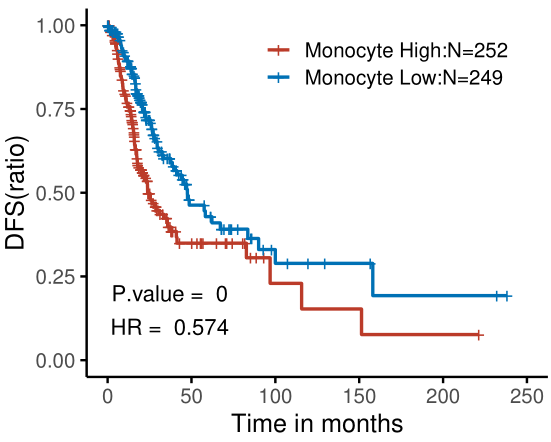

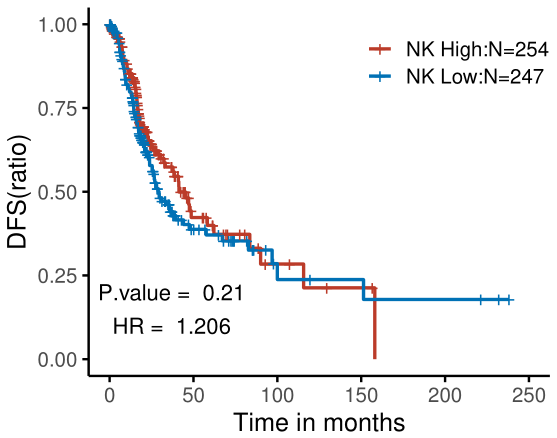


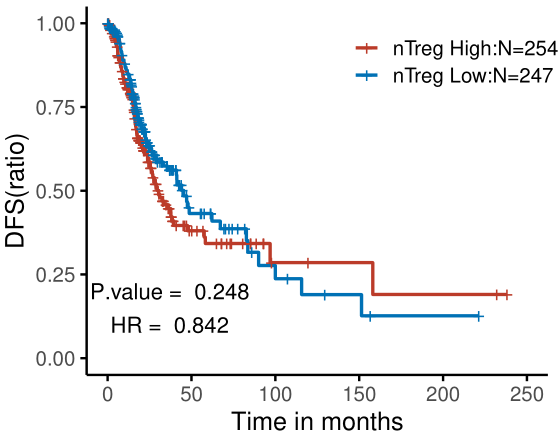

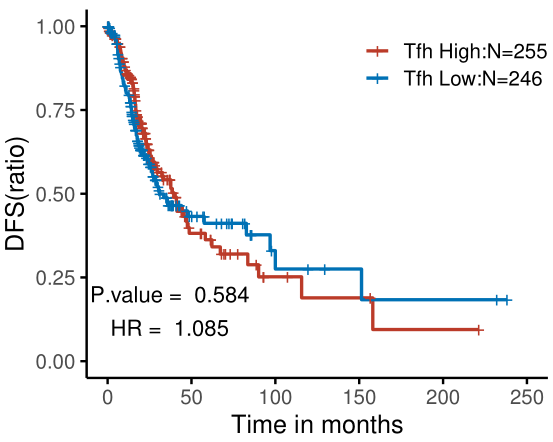


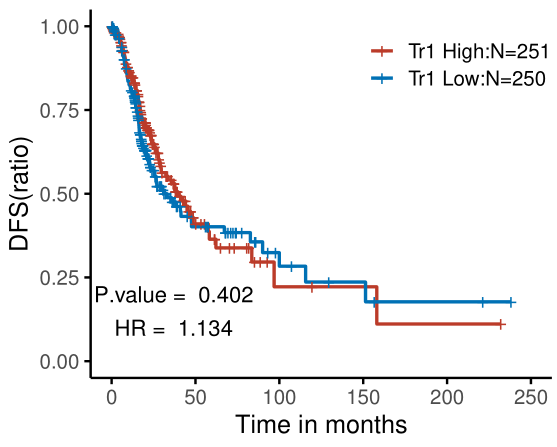


Fig.2. Analysis of the correlation between the stromal score and immune cell infiltration using the ESTIMATE algorithm.

LUAD cases were divided into high and low stromal groups according to the median value. The Wilcoxon test analyzed the correlation between stromal scores and immune cell infiltration.


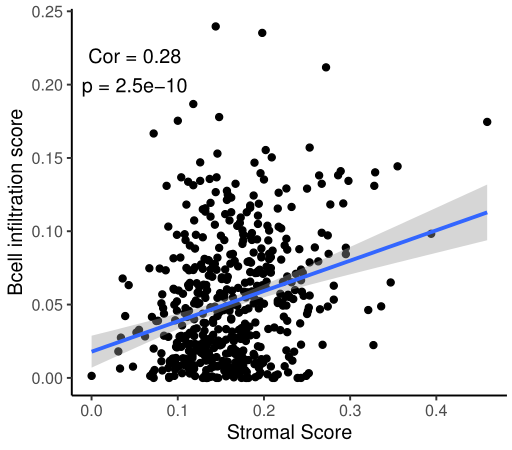

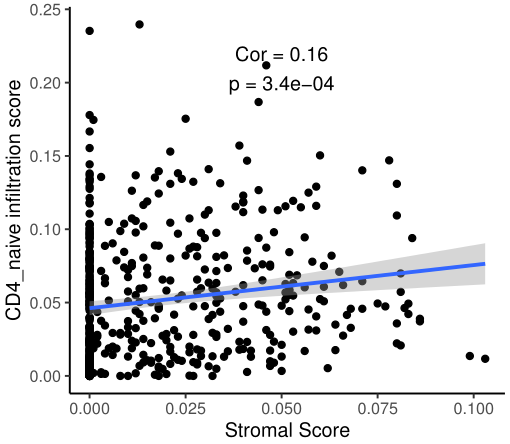

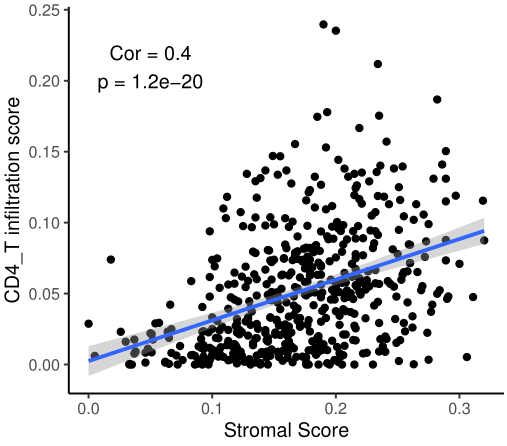

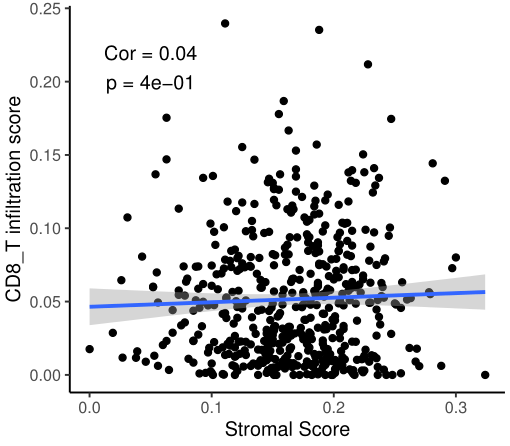

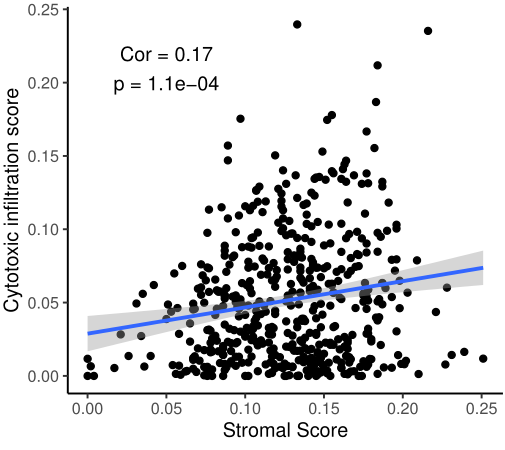

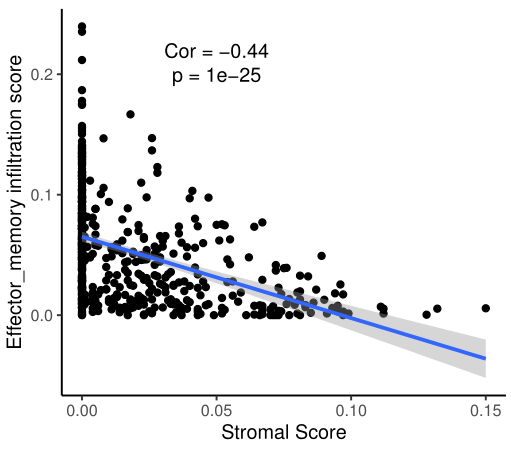

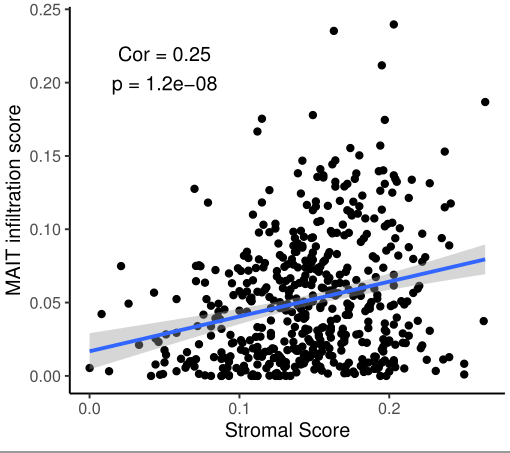

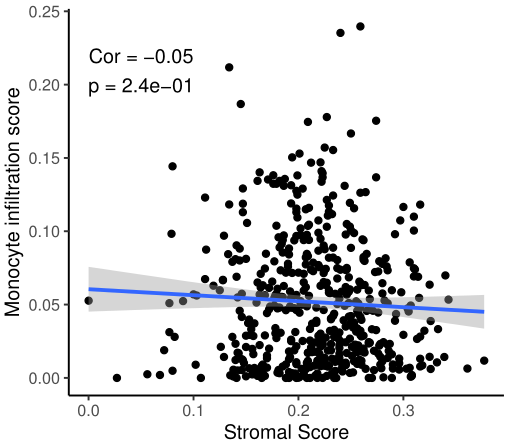


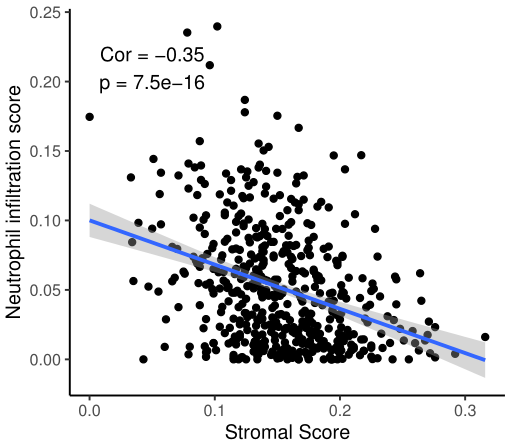

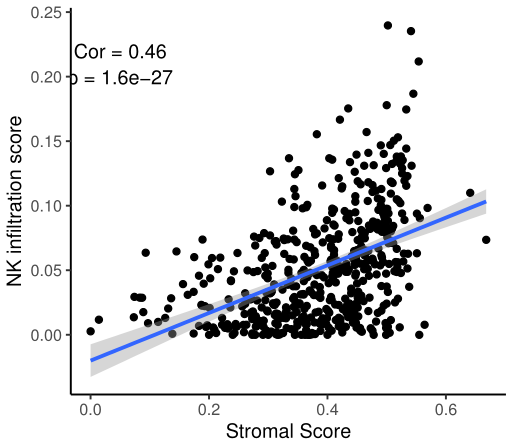


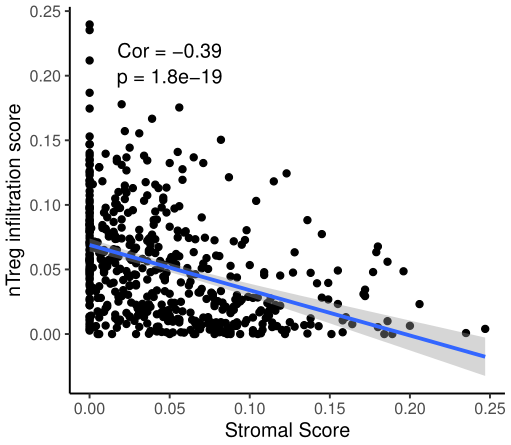

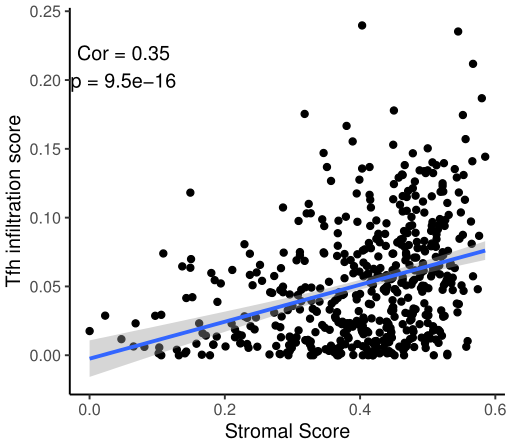

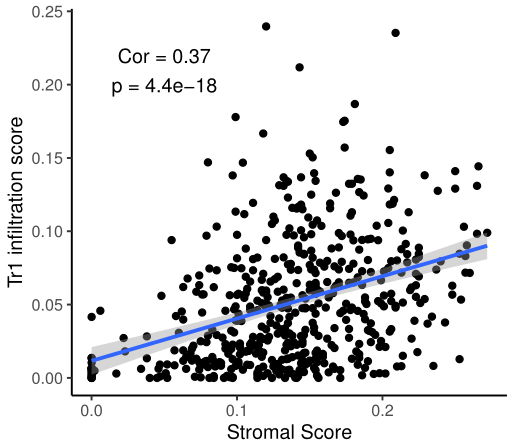


Fig.3. Analysis of the differences of immune cell infiltration in different immune subtypes of LUAD by ImmuCellAI.

The Wilcoxon test analyzed the differences between immune cell infiltration and immune subtypes, * means p< 0.05, ** means p<0.01, **** means p<0.0001.


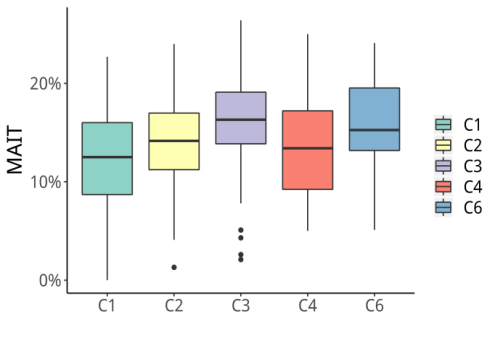

Supplement: Supplementary file 1 — Additional file 1. Supplementary material. [file 12890_2022_1902_MOESM1_ESM.docx]
